# Exploring experiences with sensitivity to cultural practices among birth attendants in Kenya: A phenomenological study

**DOI:** 10.4102/phcfm.v14i1.3322

**Published:** 2022-08-22

**Authors:** Teckla K. Ngotie, Doreen K.M. Kaura, Robert Mash

**Affiliations:** 1 Department of Nursing and Midwifery, Faculty of Medicine and Health Sciences, Stellenbosch University, Cape Town, South Africa; 2 Department of Community and Reproductive Health, School of Nursing Sciences, Kenyatta University, Nairobi, Kenya; 3 Department of Family and Emergency Medicine, Faculty of Medicine and Health Sciences, Stellenbosch University, Cape Town, South Africa

**Keywords:** skilled birth attendants, traditional birth attendants, cultural sensitivity, cultural practices, pregnancy, birth

## Abstract

**Background:**

Sensitivity to women’s cultural needs and expectations by care providers is essential. Skilled birth services for women are as essential as traditional birth services. Therefore, collaborative skilled and cultural care optimises childbearing experiences.

**Aim:**

This study explored the experiences of birth attendants (BAs) with sensitivity to cultural practices (CPs) during pregnancy and birth among the Keiyo community in Kenya.

**Setting:**

The study was conducted in the purposively selected public health centres and dispensaries offering maternity services and the villages in Keiyo South Sub County in Kenya.

**Methods:**

A qualitative interpretive phenomenological study of BAs was conducted. Iterative and inductive interviews using a semistructured guide were conducted with 11 skilled BAs (SBAs) and eight traditional BAs (TBAs). Audio-recorded interviews were transcribed and analysed using ATLAS.ti software version 8.4.4 (1135), following Van Manen’s five thematic analysis steps.

**Results:**

Three themes emerged: birth attendants’ cultural encounters, response to cultural encounters and collaboration. Birth attendants’ responses to different cultural encounters revealed their awareness of CPs. The response was experienced as a sensitivity to the need for a triad (woman, TBAs and SBAs) collaborative care, enabling collaborative, woman-centred and culturally safe care.

**Conclusion:**

Birth attendants are exposed to cultural encounters, and their responses determine their awareness of enabling sensitive care for optimal childbearing experiences. The study illuminated the need for further collaborative engagements between the BAs and the community to facilitate positive experiences by women through woman-centred, culturally safe care.

## Introduction

Every pregnancy and birth experience of a woman is different, and with each experience comes memories that she learns to appreciate or deny.^1,2^ Childbirth experiences are etched in women’s memories. The more pleasant they are, the better.^3^ The physical, social and cultural environments that women navigate influence their experiences during pregnancy and childbirth. Therefore, women’s experiences are a ‘woven fabric’ resulting from the interplay between these environments.^4^

Maternal deaths remain high. Globally, 830 women die daily. Sub-Saharan Africa accounts for 546 per 100 000,^5^ while Kenya has 362 maternal deaths per 100 000 live births,^6^ yet 70 per 100 000 live births are expected by 2030 through the Sustainable Development Goals (SDGs).^7^ Approximately 60% – 80% of them can be prevented through early identification and intervention of obstetric complications by skilled care providers.^5^ In Kenya, 95% of pregnant women attend prenatal care, but less than 40% utilise skilled birth services. These low rates of births at the health facilities are partially attributed to socio-economic and cultural factors.^8^ Women’s preferences for traditional birth attendants (TBAs) and cultural practices (CPs) remain a barrier to using skilled birth services, despite education on better pregnancy and birth outcomes when utilising skilled BAs (SBAs).

Specifically, the Keiyo rural community values such socio-cultural practices throughout all childbearing stages. Most of these CPs involve food choices, herbs and cultural practitioners’ utilisation.^9^

Although culturally competent care is recommended by professional bodies, such as the International Confederation of Midwives^10^ and the World Health Organization (WHO), and is included in the third SDG,^7^ health services have not adequately embraced such care.

Intercultural sensitivity has been defined as an ‘active willingness or self-motivation to understand and to be sensitive to the view of others from different cultural backgrounds’.^11,12^ Cultural sensitivity should be a defining characteristic of a caring health practitioner, because women prefer healthcare facilities that provide culturally sensitive care.^13^ Therefore, healthcare providers need education on cultural sensitivity to overcome the inadequate utilisation of skilled birth services by women.^14^

Collaborative care between SBAs and TBAs has been suggested and implemented in other countries through the progressive integration of both services.^15,16^ This collaboration builds partnerships that strengthen and enlighten SBAs concerning cultural care and TBAs with the necessary safety measures in caring for women.^15,17^ Although cultural issues influence this kind of collaboration, it is acknowledged that healthcare systems should also address the shortage of skilled providers and lack of partner support and disrespectful maternal care.^18,19^

According to the Kenya National Bureau of Statistics,^20^ Kenya has 44 tribes with differences and similarities in CPs. Birth attendants engage with women from these diverse ethnic backgrounds during pregnancy and birth. Women in Keiyo, Kenya, receive care during pregnancy and birth from TBAs and SBAs. Traditional birth attendants continue to provide services in the community, with no formal collaboration with SBAs and no government policy to regulate their practices.^21,22,23^

Despite the ethnic diversity in Kenya, there is limited evidence on the cultural challenges that BAs or childbearing women encounter during their engagements. Therefore, this study aimed to explore BAs’ sensitivity to CPs during pregnancy and birth among the Keiyo community in Kenya.

### Theoretical framework

The researcher utilised the cultural safety theoretical framework developed by Ramsden^24^ to guide the study (Figure 1). The framework was initially developed in New Zealand, where indigenous Maori people were affected by unequal representation in nursing schools and an increased disease burden because of discriminatory health care services.^25^ Cultural safety has been defined as:

[*T*]he effective nursing of a person/family from another culture by a nurse who has undertaken a process of reflection on their own cultural identity and recognises the impact of the nurses’ culture on their own nursing practice. (p. 491)

**FIGURE 1:**
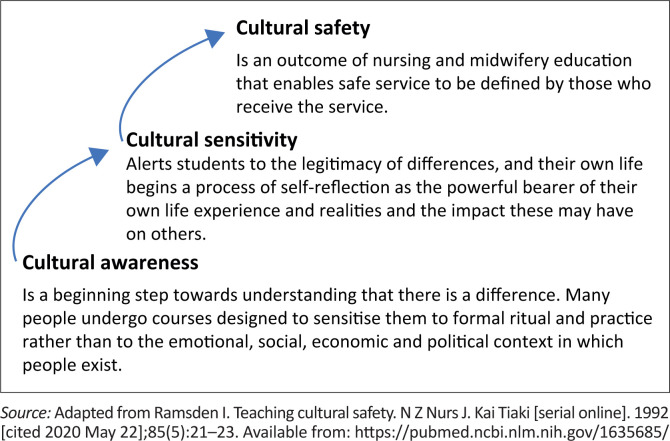
Culture safety framework.

The definition was further adapted by the Nursing Council of New Zealand to ‘relate to the experience of the recipient of care and extends beyond cultural awareness and cultural sensitivity’. Care without considering the woman’s cultural identity diminishes, demeans and disempowers the woman during pregnancy and birth.^26^

Cultural safety is considered an outcome of cultural sensitivity, requiring cultural awareness.^24^ Further, Ramsden describes cultural awareness as the conscious effort by individuals to recognise and acknowledge cultural differences. Subsequently, Ramsden asserts that cultural sensitivity is achieved through self-reflection and self-awareness, leading to the acceptance of the legitimacy of cultural differences and mutual respect. Further, Ramsden^24^ suggests the need to sensitise care providers to optimise culturally sensitive care.

## Research methods and design

### Study design

The study adopted a qualitative design^27^ and interpretative phenomenological approaches.^28^ This type of hermeneutic phenomenology explores peoples’ lived experiences about a phenomenon of interest.^28^ People’s lived experiences are identified and described, and the meaning of these experiences is interpreted.^29,30^ This design was appropriate because the researcher is a SBA, born and raised in the Keiyo community and knowledgeable about the Keiyo culture. Therefore, prior understanding of the phenomenon enabled the researcher to interpret BAs’ meanings attached to their sensitivity to CPs. The researcher was also part of the research instrument, and therefore there was no bracketing.^27,28^ However, the researcher maintained an unbiased stance throughout data collection and analysis to ensure that the findings reflected interpretations from the participants’ findings.

### Study setting

Kenya has 44 tribes, making it one of the most culturally and ethnically diverse countries. The Kalenjin tribe is the third largest tribe, accounting for about five million people.^20^ The Kalenjin are distributed mainly within the Rift Valley, with up to seven subgroups: Kipsigis (1 916 316), Nandi (949 835), Pokot (632 557), Keiyo (313 925), Marakwet (200 000), Tugen (109 906) and Sabaot (305 000). These subtribes share a common language, cultural beliefs and practices, geographical location and socio-economic activities.^31^

The Kenyan government changed the constitution in 2010 and redefined the eight provinces as 47 counties, with further subcounties.^32^ This study was undertaken in Keiyo South Constituency, a subcounty of Elgeyo Marakwet county. The population of Keiyo South is 120 750 out of a total Keiyo population of 219 750.^20^ The Keiyo, a Kalenjin subtribe, predominantly occupy Keiyo South (Appendix 1). As illustrated in Figure 1-A1,^33^ the Kerio highlands and escarpment, the hanging valley and the proper Kerio valley basin mark the Keiyo South boundaries within the Great Rift Valley (Appendix 2).^34^

There are two health systems within the Keiyo community: the Ministry of Health’s (MOH) allopathic health system and the traditional health system. The traditional health system includes the TBAs and traditional healers. Allopathic health services are provided through the MOH, faith-based and private health care providers. The MOH provides facility-based services and coordinates community health workers (CHWs) and community health volunteers (CHVs) who coordinate health services in the community health units. These units consist of 5000 people or 1000 households. They form an extension of the main health facility as service delivery structures.^35^

Dispensaries are managed by community health nurses who provide curative, promotive and preventive health services, including maternal, neonatal and child health. Clinical officers, community health nurses or midwives, public health officers, laboratory technicians and pharmacists run the health centres. These health centres act as referral centres for the community units and dispensaries. The services provided include minor surgical interventions and curative, promotive, preventive, maternity and birthing services. All these health facilities refer to the subcounty or county referral hospitals according to the severity of the cases.

The community is faced with challenges in accessing health services, especially those situated along the hanging valley and some parts of the proper valley basin because of rugged terrain and mudslides during the heavy rainy seasons. Nevertheless, with the recent introduction of motorcycles referred to as *bodaboda*, accessibility has improved. (Figure 1-A2a and b and Figure 2-A2a and b show the terrain characteristics of the study site.)^34^

### Study population and sampling strategy

The study was conducted in two phases. In phase one, five health centres and five dispensaries were purposively selected from the 36 health facilities in the subcounty. The researcher selected five facilities with an average of 20 or more births per month and five facilities below 20 births. During facility visits, SBAs on duty were approached and requested to participate. Out of the 10 facilities, one to two SBAs who had worked in maternal health units for at least six months were purposively sampled in liaison with the facility managers. This criterion enabled the inclusion of a maximum variation of SBAs, which provided data from the typical SBAs and extreme cases, especially from those who had worked for more than 20 years. An experience of at least six months was considered, as it could have exposed the SBA to the minimal basic experience with CPs in the community. An iterative interview and analysis process determined when saturation of data was obtained. Data saturation was achieved after analysing data from the first nine interviews, as the remaining two interviews did not elicit any new information.

In phase two, eight TBAs were purposively selected. Ten villages were identified with assistance from the local chief, the gatekeeper to the community. Some villages shared the same TBA. The initial TBA also assisted in identifying others through a snowballing process. The CHVs or CHWs assisted in locating the TBAs’ homes for the interviews, which facilitated the initial meeting held between the TBA and the researcher, without the CHV or CHW’s presence. The researcher and the participant agreed on the interview date and time. An iterative interview-analysis process was done. Data saturation was achieved after the sixth participant, while information from the two subsequent interviews was redundant.

### Data collection

Semistructured interviews were conducted by the researcher (first author) in 2019 for the SBAs and 2020 for the TBAs. The researcher’s personal experience and insight into this community’s cultural beliefs and practices enabled the interviews to be conducted with awareness of cultural nuances. The researcher had not worked as a SBA in this community.

A semistructured interview guide was used for SBAs, with open questions to explore the following topics: CPs in pregnancy and birth, the role of CPs in the health services and SBAs’ feelings regarding CPs during pregnancy. The semistructured interview guide for the TBAs included the following topics: TBAs’ experiences with CPs, feelings regarding CPs during pregnancy and roles of CPs during pregnancy and birth in the community.

The researcher was fluent in English, Kiswahili and Keiyo, allowing the participants to express themselves in whichever language they felt comfortable. The researcher conducted interviews with TBAs at their home and with SBAs in their health facilities, which lasted between 1.5 h and 2 h. All interviews were audio-recorded with the participant’s consent.

### Data analysis

Iterative data collection and analysis led to emerging themes on cultural sensitivity. Transcription was done from the recorded language (English or Kiswahili). Each transcript was checked against the recorded interview to ensure that the transcription was accurate. The transcribed data was uploaded into ATLAS.ti software version 8.4.4 (1135) for data analysis, and Van Manen’s five-step thematic analysis was used during the data analysis.^36^

Firstly, the researcher uploaded the transcripts to ATLAS.ti, read and reread the transcripts, which gave the researcher insight into the emerging codes.

Secondly, progressive coding resulted in identification of categories (first-order constructs). The codes were organised into code groups that were labelled as categories. The researchers reviewed the codes and categories and reached a consensus regarding the essential codes. After the iterative reading of the transcripts and listening to the audio-recordings, new codes that were identified formed other categories or merged into existing categories.

Thirdly, steps 1 and 2 were repeated for the SBA interviews by analysing three interviews, six, nine and finally, all 11 transcripts. For TBAs, two interviews, four, then six and all eight transcripts were analysed. The analysis of initial transcripts identified issues that required further exploration in subsequent interviews and the creation of code categories. Related code categories were merged according to the emerging subthemes. This iterative process gradually developed a ‘richer and deeper understanding’^28,29^ of the BAs’ experience with sensitivity to CPs as subthemes emerged from the ATLAS.ti-generated report and networks that elicited relationships in the subthemes.

Fourthly, second-order constructs, which were the emerging subthemes, were therefore merged into overarching themes, which were determined and agreed upon with the first author who had insight into the phenomenon under study. ATLAS.ti-generated reports and networks, which had all the quotations under each code, were used for interpretation.

Finally, the codes were summarised into categories which formed the subthemes and overarching themes presented in Table 3. Refining of the themes and interpretation of the meanings ascribed to sensitivity were further interrogated during the article writing process through consensus amongst the researchers. Findings are presented as an interpretation of themes, actual quotes from the interviews, integrating interrelated themes with cultural safety framework (Figure 2)^24^ and drawing on input from the literature.^27,36^

**FIGURE 2:**
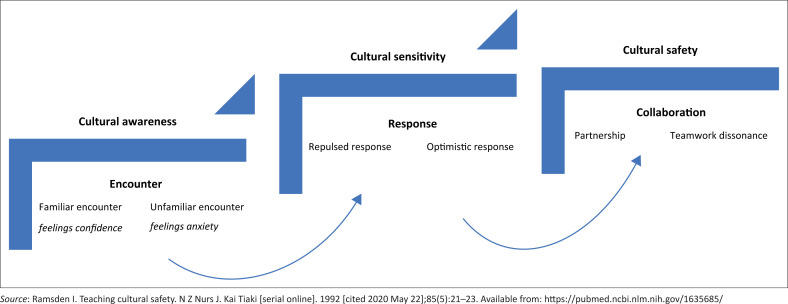
Interrelated themes incorporating cultural safety framework.

### Ethical considerations

Ethical approval was granted by Stellenbosch University Health Research Ethics Committee (ref. no. S18/10/255) on 26 April 2019, and after that, renewal and extension of the permission were granted on 02 November 2020. Following the HREC approval, the country’s approval was granted by the Africa Medical and Research Foundation (AMREF) Ethics and Scientific Review Committee (ref. no. P642/2019) on 27 June 2019, with approval of extension granted on 26 November 2020. The National Commission for Science, Technology, and Innovation (NACOSTI) ref. no. (NACOSTI/P/19/34135/30641) further approved the research to be conducted in Kenya. Permission to access the SBAs in the county was granted by Elgeyo Marakwet County and facilities’ administrators. In contrast, permission to access the TBAs was given by the chiefs, who are the juristic persons in the community.

## Findings

### Characteristics of participating birth attendants

Eleven SBAs were interviewed; five were male and six were female. They were between the ages of 23 and 55 years. All participants were fluent in both English and Swahili languages. They originated from the various Kalenjin subtribes, of which six were from Keiyo, three were from Tugen and two were from the Marakwet subtribe. Their work experience in maternal and child health ranged between one and 30 years, as indicated in Table 1. Ten of the participants had a diploma qualification, while one had a bachelor’s qualification.

**TABLE 1:** Characteristics of the participating skilled birth attendants.

Characteristics	Number
**Age (years)**	
20–30	4
31–40	5
41–50	1
51–60	1
**Gender**	
Male	5
Female	6
**Subtribe**	
Keiyo	6
Tugen	3
Marakwet	2
**Years of employment**	
1–10	9
11–20	2
**Highest qualifications**	
Bachelors	1
Diploma	10

A total of eight TBAs were eligible to participate in the study. All eight TBAs were female, between 45 and 72 years, and they had between 10 and 40 years of experience. All eight were fluent in Keiyo; three of them also had a moderate understanding of Swahili, and three were fluent in Swahili. The TBAs were from the various Kalenjin subtribes: five from Keiyo and one from Marakwet, Kipsigis and Tugen (as indicated in Table 2).

**TABLE 2:** Characteristics of the participating traditional birth attendants.

Characteristics	Number
**Age (years)**	
40–50	1
51–60	2
61–70	4
71–80	1
**Gender**	
Male	0
Female	8
**Subtribe**	
Keiyo	5
Tugen	1
Marakwet	1
Kipsigis	1
**Years of experience**	
10–20	3
21–30	3
31–40	2

### Themes on birth attendants’ sensitivity to cultural practices

Three interrelated themes emerged through exploring BAs’ experiences with sensitivity to CPs during pregnancy and birth (Table 3). They are further described in the following subsections.

**TABLE 3:** Themes for birth attendants’ sensitivity to cultural practices.

Themes	Subthemes
Encounters with cultural practices	Familiar encounters Unfamiliar encounters
Responses to cultural practices	Optimistic responses Repulsed responses
Birth attendants’ collaboration	Partnerships Teamwork dissonance

#### Encounters with cultural practices

Birth attendants recounted how they felt when they encountered a CP. Their encounters with CPs were conceptualised as either familiar or unfamiliar, as determined by the feelings evoked, such as fear, anxiety or fulfilment. Those BAs familiar with the CPs felt more confident in their care. This confidence made them feel that they provided sensitive care. However, some BAs expressed that an unfamiliar encounter with CPs triggered fear and anxiety. They were worried about the poor outcomes that might result from their care when they were unfamiliar with the CP.

### Familiar encounters

Familiarity with encounters was based on whether the BAs had prior experience with the CPs. Such experience was common for TBAs. However, SBAs’ familiarity with CPs was often because of their affiliation with a particular tribal origin. Because of their familiarity with the CPs, the BAs felt confident and in control, and they anticipated favourable outcomes for the childbearing women, caregivers and community. However, the familiarity did not necessarily mean that the birth attendants were entirely comfortable with the encounter, and some anxiety could still be felt.

One TBA narrated an experience where she encountered a mother with a swollen breast 5 days after birth. The TBA was familiar with this problem and felt comfortable and assured that she could handle the situation successfully:

‘… There was this woman whose breast was swollen after getting the baby; I was summoned … when I reached there, she was sitting down with no shirt … then I cut the breast to relieve the swelling; the breast was shiny. She was in pain … at once, I asked for water to be boiled. I sent a boy to the shop to buy a razor blade … I cut along a swollen vein and drained it out. By the time I left, she was eating her food …’ (TBA 5, years of experience [E26], age [A65])

On the other hand, for SBAs, CPs that aligned with or complemented biomedical practices were experienced as familiar. For example, SBAs were familiar with the cultural value that women attach to having a companion (often a TBA) during their visit to the facility. Male companionship was also experienced as occurring more often now, as opposed to previous cultural expectations. The SBAs were confident that the companionship of women enabled woman-centred sensitive care:

‘… For me, I see them [*companions*] escorting the mothers to the facility, especially the females, and helping by providing her [*the woman*] with food, making calls to update the relatives … dressing the baby … male partners never used to come here [*maternity*], but some do nowadays, which is good.’ (SBA 6, male [M], A35)

Additionally, SBAs encountered female genital mutilation (FGM) and dietary practices as familiar CPs because they were cognisant of the implications of these practices on pregnancy outcomes. For example, one SBA narrated their concern regarding a familiar CP of withholding protein foods for fear of birth complications. The SBAs were aware that the lack of proteins could lead to anaemia and therefore were prepared to provide haematinics to the women. Further, FGM and complications such as narrowing the birth canal, which led to bleeding because of tears, were encountered as familiar. The midwives would prepare episiotomies to ensure women received the care they needed:

‘On food issues, they [*women*] come here, and when you assess them, they are anaemic. Why? Because culturally, they are not allowed to eat proteins. They [*the community*] should know that proteins are the most essential at this time [*pregnancy*].’ (SBA 3, M, A33)

‘FGM mostly affects childbirth because it causes the birth canal to narrow down due to the scarring tissue around … so it causes perineal tears, or they [*women*] are given some episiotomy that still causes bleeding to some extent.’ (SBA 6, female [F], A30)

### Unfamiliar encounters

Birth attendants usually anticipated some level of control in the care they provided to women to optimise outcomes during pregnancy and birth. Their encounters with unfamiliar CPs caused discomfort because of the possibility of losing control and poor outcomes during pregnancy and birth. In this situation, BAs felt that they were more culturally insensitive.

Birth attendants expressed a lack of confidence in an encounter that was unfamiliar to them. Further, the unfamiliar encounter evoked fear and anxiety, which exacerbated the lack of confidence. A SBA felt unfamiliar with cultural beliefs about FGM’s effects on libido and was uncertain about how to respond. On the other hand, a TBA was unfamiliar with how to handle a complication of labour, such as malpresentation, and was afraid of a poor outcome if they did not respond appropriately:

‘First, it [*FGM*] changes [*external genitalia*]. It’s no longer the anatomy as in the original normal anatomy. So, one, it [*FGM*] affects the mother during childbirth. Secondly, which I am not very much sure about, they say it affects the libido. That one, I am not sure, but I have heard that FGM to the mother affects her libido, and those are the things I wonder.’ (SBA 3, M, A33)

‘When I was still new [*to TBA services*], the thing that scared me most was when the baby’s legs came first. The grandmother [*older TBA*] pulled the baby and sucked the mucus with her mouth, then sprinkled some “tobacco” [*cigarette powder*] in the nose … the baby started sneezing and crying … I had never seen such things …’ (TBA 5, E26, A65)

Birth attendants interpreted cultural encounters based on how they felt about them. Further, the encounters enabled them to reflect on their actions and how these actions enabled their sensitivity. The anticipated outcomes of the encounters influenced their feelings of either confidence, fear or anxiety.

#### Response to cultural encounters

The BAs’ responses to encounters as either repulsed or optimistic were based on the encounters’ feelings.

### Optimistic responses

Birth attendants expressed their response to CPs with optimism if they handled them comfortably, meaning that they were hopeful that the practice could be changed or incorporated into their approach to care and were more accepting of it.

They also had an optimistic response when they saw the potential for the woman or other BA to change their behaviour and be more closely aligned to their perspective on what was appropriate. In general, optimistic responses were regarded as culturally sensitive to familiar or unfamiliar CPs.

For example, one TBA narrated how she wished the SBA could allow the woman to walk around the ward or let the TBA be with her to guide her progress during birth. Although the SBA did not allow this, the TBA was optimistic that all was not lost, as her presence in the facility enabled her to provide support to the woman by sneaking in porridge to ensure the woman had some strength during labour:

‘Imagine the mother labouring for many hours, and she is instructed to lie on one side since we arrived here last night. I did not even eat anything myself. I just sat there, but at least I sneaked in “uji” porridge in the morning … you see, if she could have come out and walked around or even kneel in a place like there [*pointing a spot with grass*], that baby could have been born “kitaaambo” [*looong time ago* …].’ (TBA 2, E25, A68)

Another example from the perspective of the SBAs was related to the use of herbs. While the SBAs did not support the use of herbs, they realised that the CP was part of the community’s culture and expressed optimism that with education and advice, the practice would die off or the complications would be averted. The SBA considered that this response was culturally sensitive:

‘You cannot do away with CPs of a community per se. However, there are some which health education should shed away, especially the use of herbal medicine during pregnancy.’ (SBA 4, F, A46)

Traditional birth attendants were optimistic when a CP such as taking herbs led to a positive outcome, and they experienced this as culturally sensitive care. For example, a TBA related how she was successful in helping a woman conceive after many years of visiting the hospitals and traditional healers unsuccessfully. She was optimistic that if such practices were allowed, many women would have good reproductive outcomes:

‘She [*woman*] came after going round the hospitals and everywhere seeking to conceive, but all was in vain. She told me that she used these “family” [*family planning*] [*contraceptives*] since she was in school [*college*] … now I knew all the veins [*fallopian tubes*] were blocked and needed herbs to “ngiriir” [*milk out the blocking substances*]. She did not even take the herbs for 2 months before she conceived … and you know that you cannot treat the woman alone. The husband must also be cleaned, because he might be the one whose veins [*tubes*] were blocked. So I gave herbs to both. As we speak now, they have two girls … yeah, you see …’ (TBA 1, E22, A58)

Skilled birth attendants also had optimistic responses to women they perceived as suffering the consequences of CPs which were not their fault. One SBA narrated an example of FGM which was not the woman’s choice and which therefore required a more sensitive response. Further, teenage abortion was forced on girls, who ended up with infections that were not their fault and needed to be treated more sensitively. This encounter led to an optimistic response from the BA, as they felt it was not the woman’s choice to get the abortion, but rather the teenage girl was coerced into aborting the baby:

‘When they [*women*] come, you don’t show them that it [*FGM*] is not permitted, or it is bad. Rather, you talk to her positively, and you show that you accept her the way she is because you cannot reverse the action, or she may have been forced to do it [*FGM*]. So you don’t harass.’ (SBA 10, F, A28)

‘There was this girl in Form 2 [*year 2 in secondary* or *high school*] who came with PV [*per vaginal*] bleeding. She was sceptical to answer questions. But I used my tactics, and finally, she told me that her mother took her to another “gogo” [*grandmother*] who put something in her vagina and told her to go home and lie on the bed until when the pain is unbearable or if she sees blood coming out [*PV*], then rush to the hospital. Imagine, she was told not to mention anyone’s name or else … so I imagined how she was feeling. So we just managed her like she was in real labour until she was strong enough to go home …’ (SBA 6, F, A30)

Skilled birth attendants saw optimistic responses as not necessarily doing away with CPs, but accepting the status quo or providing health education to change practice. The TBAs felt the need for the SBAs to alter their management of women, particularly during birth, and felt optimistic when they had the opportunity to influence the situation.

### Repulsed response

Birth attendants felt repulsed by an encounter that made them feel uncomfortable. In this situation, SBAs compelled women to act in a manner acceptable to them and disregarded other models of care. Likewise, when seeing a woman in the community, the TBA would reject the SBA’s recommendation and insist on the woman continuing with the herbs, as she felt the woman should not turn away from her tradition:

‘It is not good to run away from your “mila” [*tradition*]. You [*women*] go to the doctors but do not stop taking your herbs every morning. She brings the doctors’ [*SBAs’*] report to me and reads it … for us old women, we don’t know how to read those reports, but if she [*woman*] says that everything is OK, then we continue watching out.’ (TBA 2, E38, A70)

On the other hand, SBAs felt repulsed by specific CPs related to the TBAs’ care, such as encouraging women to have homebirths or delaying referral when there was a complication. Likewise, the SBAs felt disappointed when the TBAs would contradict their advice or instructions:

‘Sometimes, these TBAs, if they are encouraged [*to do home births*], they wait for so long when there is a problem … imagine, these pregnant women attend all the required clinics, and when the last period of giving birth comes, they do at home because they are swayed by TBAs …’ (SBA 4, F, A46)

‘So you say a point [*advise the woman*]; they [*TBAs*] say leave that [*to the woman*]. I mean, they do the opposite of what you are saying, or if I tell the mother not to push without an urge, she [*TBA*] tells her to push! Push … you tell someone to push at 6 cm [*cervical dilation*]. What are you doing? If the mother has not told us [*SBAs*] that she has the urge, why should you tell us that we are inexperienced?’ (SBA 7, F, A30)

Although SBAs recognised that they could not stop women from using herbs, they attempted to persuade women to postpone using herbs to minimise complications during labour. The SBA felt that there was no way of stopping the women from using the remedies, as they were caught in this mindset of using remedies during pregnancy and birth. In particular, the SBAs felt repulsed by educated women who continued using herbs. For example, one SBA recalled a university student who had a herb-induced abortion. She expected the girl to seek long-term family planning methods rather than becoming pregnant and then using herbs to induce an abortion:

‘They are stuck with their herbal remedies; for me, I have been trying to inspire them to delay giving herbal medicine because I am part of them. I cannot refuse them to practice them, but we postpone. The moment we postpone, we minimise the complications.’ (SBA 2, M, A32)

‘You are even an educated person; why do such things [*herb-induced abortion*]? Nowadays, there are a lot of family planning methods. You could have just gone to any facility, and they would provide a long-term method for 5 years; then the only thing you worry about is HIV.’ (SBA 6, F, A30)

Traditional birth attendants expected women to comply with CPs and abide by their advice during pregnancy. However, TBAs were repulsed by women who failed to adhere to their cultural expectations. A repulsed response was noted when a TBA spoke about a woman who continued having sexual intercourse during pregnancy and a woman who did not take the herbs for *katet* [sharp lower abdominal pain]:

‘These young women don’t listen! Like this one woman who came to me in the morning saying that she did not sleep last night because of pain on the lower abdomen around here [*rubbing around the symphysis pubis*]. I just knew it was *katet* because these girls continue encouraging their men to sleep with them [*having sexual intercourse*], pwai! Ptu! [*Disgust exclamation while spitting out saliva*] …’ (TBA 2, E35, A68)

‘I tell you! My child, it is hard dealing with these women sometimes. The other day before Christmas, a woman came here [*TBA’s home*] feeling pain in the abdomen. I touched [*abdominal palpation*] and found out that the baby was lying well, but the mother had *katet*. I gave her some herbs to boil and take. Well, after about 2 weeks, she came back complaining of the same problem. When I asked her if she had taken the herbs, she said no. She had gone to the hospital for the same problem, but you know, hospital medicine does not treat *katet*. Why was she coming back to me if she did not take the herbs? Hmm …’ (TBA 1, E40, A70)

Skilled birth attendants’ responses were often about their biomedical practice, while TBAs’ responses were about what they considered appropriate as per their CP. The woman’s perspective might be overlooked in both situations, leading to a culturally insensitive response. Repulsed responses by BAs were more likely to be culturally insensitive.

#### Collaboration

Some BAs felt that a more woman-centred model of care required collaboration between SBAs and TBAs, who were both vying for the woman’s agreement with their plan of action. According to the BAs, health services that strive to be more woman-centred might encourage partnerships and avoid teamwork dissonance among BAs and other stakeholders within the health care environment. Such a model might be considered most sensitive to women’s cultural needs and expectations.

### Partnerships

The partnership is about creating an organisational environment that supports joint actions between SBAs, TBAs and other stakeholders in supporting women during pregnancy and birth. Such an environment requires integrating both biomedical and traditional models of healthcare.

These partnerships could enable appropriate responses to CPs that provide woman-centred care. For instance, one TBA appreciated the enlightenment they received from World Vision, a nongovernmental organisation, which educated them on the danger signs to look out for during pregnancy and birth. Further, the TBAs were rewarded by World Vision when they referred the high-risk women to the SBAs:

‘World Vision really helps us here [*community*] … they show us how to look out for danger signs, and we send them [*women*] to the hospital … they give you something small [*token*] if you refer them [*women*] ….’ (TBA 5, E26, A65)

Such a partnership model would support mutual respect between SBAs and TBAs to enable women to make informed and shared decisions about their care. Women would remain in control of their health care and make informed and mutually agreed-upon choices without coercion, ordering or instructing.

Skilled birth attendants narrated that, occasionally, they gave gloves to the TBAs and would encourage them to accompany women to the health facility. Although there are no clinical policies and guidelines that recommend the supply of protective equipment or the involvement of TBAs, the SBAs felt the need to enable this type of partnership to provide safe care:

‘OK, we have embraced the issues … I mean the issue of TBA, by teaching about pregnancy and childbirth. Also, we have been distributing gloves, and we have encouraged them to come to the facility if a problem arises. We do not normally chase them. We encourage them to accompany the mothers to the facility. We usually talk to them nicely. We don’t harass them and tell them, “Why did you deliver … I mean, why did you accompany the mother?” We usually embrace and support them.’ (SBA 7, F, A30)

‘*Madaktari* [*healthcare workers*] help us sometime by giving us gloves to use in case it [*birth*] happens in your hands … you can’t refuse [*conduct birth*] when she’s here, and you can see the baby coming … heheeii … *ichurchini* [*you dare*] and hope that God will protect you … the next day, you go with her to the midwife *na ujistaki* [*and report your actions*].’ (TBA 3, E25, A55)

According to birth attendants, partnership implied a strategy for organisational structures to support woman-centred negotiated care within the triad of TBAs–SBAs–women as they encountered CPs. Fostering relationships through sharing resources, providing free maternity services, involving mother-mentors as trailblazers of women’s support groups and incentivising TBAs to refer women for SBA services were all seen by BAs as being sensitive to women’s needs. However, there was no consistent or sustainable approach at present to developing such partnerships.

### Teamwork dissonance

Teamwork emerged when most BAs made a concerted effort to foster a triadic team (woman–SBA–TBA) that would communicate what women needed to know about their care and agree on a plan. Traditional birth attendants hoped the SBAs could appreciate their experience with CPs, while the SBAs expressed the need for the TBAs to acknowledge their expertise. Therefore, BAs envisioned a situation where the community supported the triad by promoting safe CPs and involvement in public health forums and campaigns.

The TBAs were apprehensive of being removed from the birthing rooms while supporting the women, assisting with the baby and communicating with the relatives:

‘When I go with the mother, some doctors [*SBAs*] will allow you to sit in the same room where the mother is labouring … Some [*SBAs*] do not chase you, but they take her in [*birthing room*]. I will be waiting close by with clothes for the baby. As soon as I hear the cry, they allow me to go and dress the baby while she [*SBA*] finishes up with the mother. It feels good to be of help there [*facility*] … my work is to dress the baby and escort the mother back to bed … I will rush to the shop to get her tea and bread to warm herself with. Then I will inform the people at home that they are *sere* [*blessed*]. Good thing we have their phones that you can call and tell them [*relatives at home*] to bring porridge and other things that we need …’ (TBA 5, E26, A65)

From the findings, not all SBAs appreciated engagement with the TBAs. Skilled birth attendants suggested a forum whereby TBAs and SBAs could meet to discuss and share expectations and provide updates on better maternal care acceptable to both BAs and the women. The SBAs felt that it had reached a point where the TBAs are competing with SBAs, which leads to complications such as postpartum haemorrhage and puerperal sepsis:

‘Just insist on if we can look for ways of how to break that gap between the TBAs and the health workers. Suppose there is a way to set a meeting on how we can support safe deliveries as one thing [*pause*], ’cause I see that it has reached a point. In that case, it is like we are doing a competition, but despite the competition, we get more sepsis; we get more PPH [*postpartum haemorrhage*] … then they come here and say, we just made it halfway and the baby came … such things. You get issues with the placenta, PPH and other funny, funny things.’ (SBA 7, F, A30)

Inadequate or lack of teamwork emerged as the leading cause of friction between the BAs, which caused unsafe care practices for the woman. The suggestion of involvement of the triad in solutions that promoted safe practice could eliminate the dissatisfaction among the parties. For example, the TBA felt unhappy when the SBA ignored her plea to assist the woman during labour and felt dismissed even though she was sure the woman needed her help:

‘She [*woman*] was starting to push. I went there [*SBA station*] and told her that “I think she is ready” [*to give birth*]. She [*SBA*] did not even want to listen to what I [*TBA*] told her. She [*SBA*] just dismissed me and said, “I know what I am doing.” I just went back only to find the woman giving birth. Then I shouted, “please come!” while I was preparing to assist her [*woman*] … you see these people [*SBAs*] …’ (TBA 6, E15, A62)

Collaboration needed to be built on mutual respect where teamwork was encouraged rather than ignored. It emerged that the TBAs expressed expertise in culturally appropriate care after attending traditional ‘schools’ where they were mentored by a traditional midwife, who would hand over the practice once she was convinced that the mentee would not embarrass her. The TBAs viewed this as equivalent to the education the midwives received:

‘Those midwives went to school to read on childbirth issues. Even us, we went to ours, so we have traditional education because when you perform by the side of a well-experienced *Korgob sigisio* [*traditional midwife*], you become even better than her … she holds your hand [*mentorship*] until she gives a hand to you [*handing over the practice*] when you are ready and will not embarrass her [*TBA mentor*] …’ (TBA 5, E26, A65)

The ‘tug of war’ between who had better education and expertise between the BAs shows the need for negotiated teamwork with clear roles agreed upon between the triad.

Teamwork dissonance could be avoided by sensitising the community to CPs that are not aligned to the biomedical model and are considered harmful. The SBAs acknowledged their role in understanding the CPs within the communities deployed. This approach emerged to enable the triad to engage with valuable and harmful practices and agree on what is feasible:

‘The only way that we will incorporate [*CPs*] as health workers is that when you [*SBA*] are posted in a certain community, it is good to know their culture and taboos, then you do sensitisation … Some practices cannot align to current health care status … you will also try to weigh the positives and negatives of that culture.’ (SBA 11, M, A55)

## Discussion

Figure 2 summarises the key findings relating to Ramsden’s cultural safety framework.^20^ Birth attendants were aware that their encounters with CPs led to emotional reactions, depending on how familiar the BA was with the practice. These reactions influenced how the BA responded and their cultural sensitivity. More sensitive responses were seen as optimistic and more insensitive responses as repulsed. Further to enable cultural safety, reflective learning from the encounters and responses enabled the BAs to identify team dissonance as a problem and appreciate efforts for partnership and woman-centred culturally safe care. Birth attendants suggested the need for all stakeholders to create partnership (women, TBAs, SBAs, community, MOH and nongovernmental agencies) and enable collaboration in the triad (SBA–woman–TBA), the team most directly responsible for culturally sensitive care.

**FIGURE 3:**
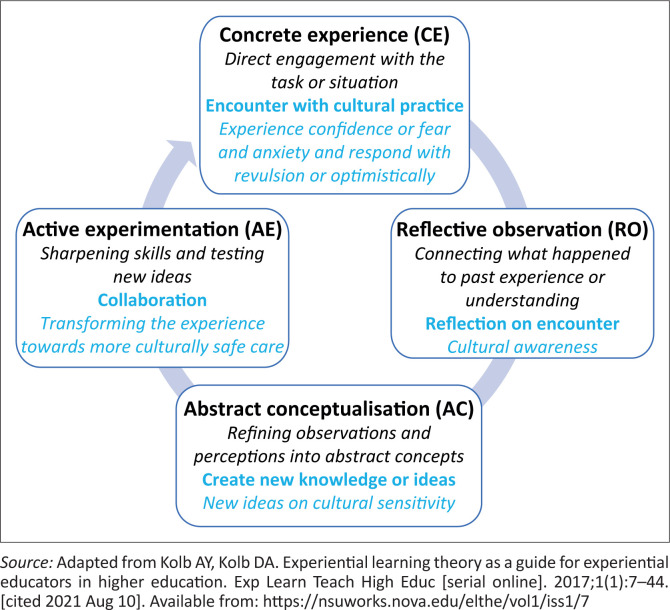
Applying Kolb’s experiential learning cycle to the development of more culturally safe care.

Achieving this ideal of collaborative woman-centred care emerged during data analysis as dependent on the ability of BAs to reflect on their experiences and learn from them. This cyclic experiential learning by the birth attendants can be viewed through the lens of Kolb’s experiential learning theory as illustrated in Figure 3 (KELT).^37^ Birth attendants’ reactions to the encounters induced feelings of confidence or fear and anxiety, and the experience could evoke a repulsed or optimistic response. After reflecting on the encounter and their response, the researcher observed that the BAs could learn and create new self-knowledge about their cultural sensitivity. This new knowledge emerged in the data, influencing their approach to future encounters and enabling a more collaborative practice.

Kolb asserts that learning is a process whereby new knowledge creation is through experience and transformation of that experience.^37^ During the initial stage of concrete experience (CE), there is direct engagement with the task or situation. In this study, the BAs had encounters with CPs during active involvement with the women, which evoked the experience of confidence with familiar encounters or fear and anxiety with unfamiliar encounters. In the second stage, reflective observation (RO), Kolb asserts that the individual connects with past experiences or understanding and reflects on their experience. This study highlighted that the cultural encounters compelled the BAs to reflect on their feelings and responses.

The reflections lead to abstract conceptualisations (AC), where Kolb highlights that reflections are refined into abstract ideas. The BAs, during the interviews, reflected on their responses to the encounters, and this awareness led to new ideas or insights into cultural sensitivity. Therefore, evaluation of the encounter led to an ‘updating’ of their prior knowledge or viewpoint.

Acquisition of this new knowledge enabled BAs to plan more collaborative approaches to care within a broader commitment to partnership with key stakeholders. Putting these plans into action and testing new ideas are highlighted in Kolb’s cycle as active experimentation (AE). This collaborative approach emerged in the study as enabling woman-centred and culturally safe care. Such responsiveness to people’s perceived health needs results in better health outcomes.^38,39^

Therefore, creating cultural awareness is more than being familiar with CPs, but it requires engagement with a discourse to understand oneself. Cross-cultural representation requires self-reflection and historical awareness.^40^ Cross-cultural engagement also requires negotiated and clear communication with people of diverse cultural backgrounds^41^ and reflecting on interactions with other culturally diverse groups builds a better understanding of ways to engage them.^42^

Engaging in self-reflection could challenge cultural norms that label people from other cultures as ‘weird’ or ‘abrasive’.^42^ Birth attendants needed to move from judging others based on their normative viewpoints to accepting the legitimacy of other viewpoints, even if they disagreed with them. It emerged that their responses were concerning their concept of self-versus the other.^43^ Developing such cultural competency is a crucial proficiency in social work and midwifery.^40^ Collaboration emerged as an area for AE by BAs. However, experimenting requires self-efficacy.^43^ The foundation of collaborative engagement is shared decision making between the recipient and provider of care.^44,45^ Shared decision making between the BAs was appreciated, while team dissonance was viewed as the reason for poor outcomes during pregnancy and birth.

Further, there is a need for the concept of partnership to be included in guidelines and policies that set direction and support for team-based approaches to care.^46^ These organisational processes build a shared understanding between partners and build relationships within the team. Birth attendants identified the need for practice regulation to create such partnerships and avoid team dissonance.

### Strengths and limitations

All BAs were from the Kalenjin tribe and shared a similar cultural background. The findings were interpreted based on the specific characteristics of the participants within their context. The transferability of the findings will depend on the participants and the study context in a different study.

Interpretation of data depended on the researcher’s philosophical positioning. However, the researcher ensured that preconceptions of cultural and professional affiliation did not significantly influence the interpretations of the data. Supervisory guidance and reflections assisted in the interpretive character of understanding the BAs’ experiences.

Limitations were related to methodology during data collection and analysis, which was time-consuming and tedious. But some of these were mitigated by involving a transcription assistant and the use of ATLAS.ti software for coding.

### Recommendations

From the findings, SBAs need to develop cultural sensitivity, and continuing professional development should focus on this. Such education should facilitate experiential learning, critical thinking and reflection amongst midwives. Reflective learning on cultural sensitivity and culturally safe care should also be embedded in midwifery curricula. Trainers should also embody such cultural sensitivity.

Maternal care guidelines should explicitly address cultural sensitivity and safe care and promote an organisational culture that supports partnerships. An approach to collaborative practice that includes TBAs as part of the team will reduce women’s feeling of giving ‘birth in between’ modern and traditional health settings.

Further studies should focus on developing such educational interventions and testing their effectiveness. The implementation of educational and organisational change should also be evaluated.

## Conclusion

Birth attendants reacted to encounters with CPs with various feelings such as anxiety or confidence. These reactions appeared related to whether the BAs experienced a familiar or unfamiliar encounter. The response to these encounters was observed as being either repulsed or optimistic. Further reflection on the encounters illuminated the need for the BAs to collaborate through teamwork and develop the necessary cultural awareness and sensitivity. In addition, the health services needed to create a commitment to partnership between modern and traditional stakeholders to enable acceptable and woman-centred care. The development of cultural sensitivity and safe care may require reflective learning, supportive guidelines and collaborative practice, particularly between SBAs, TBAs and the women under their care.

## Figures and Tables

**FIGURE 1-A1:**
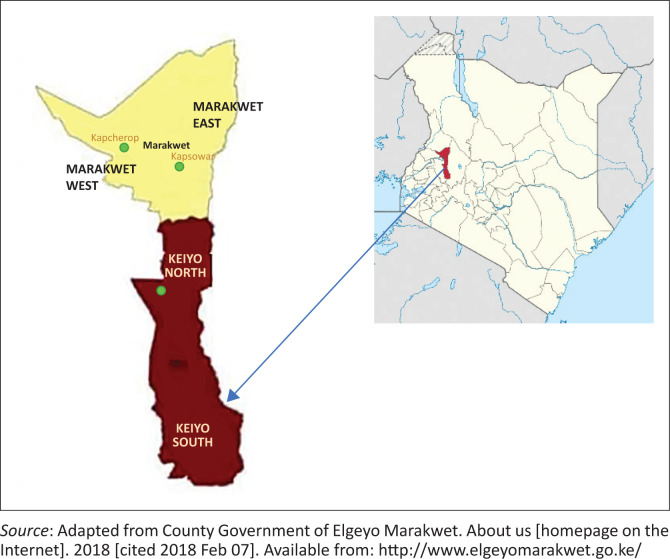
The map of Kenya, showing Elgeyo Marakwet county and Keiyo South subcounties.

**FIGURE 1-A2:**
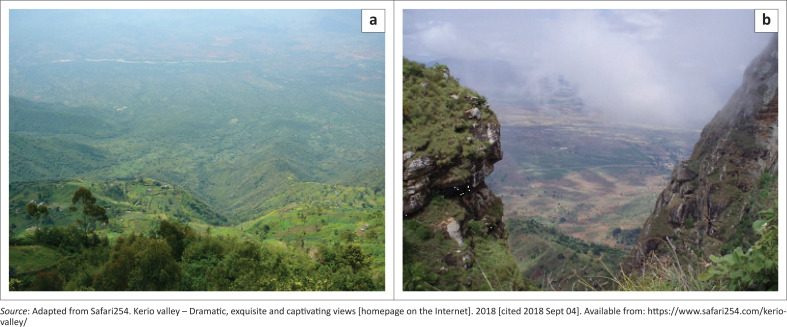
(a and b) Characteristics of Keiyo South subcounty terrain.

**FIGURE 2-A2:**
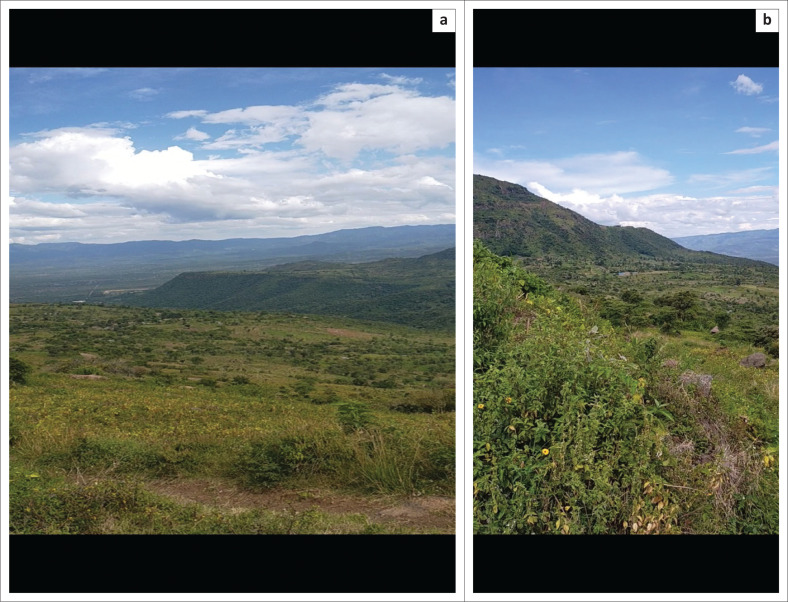
(a and b) Characteristics of Keiyo South subcounty terrain.
